# Inducible nitric oxide synthase-expressing myeloid-derived suppressor cells regulated by interleukin 35 contribute to the pathogenesis of psoriasis

**DOI:** 10.3389/fimmu.2023.1091541

**Published:** 2023-03-09

**Authors:** Junfeng Zhang, Yunsheng Zhang, Zhiya Yang, Dalei Cheng, Hui Zhang, Li Wei, Chen Liu, Fenglian Yan, Chunxia Li, Guanjun Dong, Changying Wang, Dongmei Shi, Huabao Xiong

**Affiliations:** ^1^ Institute of Immunology and Molecular Medicine, Jining Medical University, Jining, China; ^2^ Jining Key Laboratory of Immunology, Jining Medical University, Jining, China; ^3^ Department of Dermatology & Laboratory of Medical Mycology, Jining No. 1 People’s Hospital, Jining, Shandong, China; ^4^ Affiliated Hospital of Jining Medical University, Jining Medical University, Jining, China

**Keywords:** imiquimod-induced psoriasis mouse model, inducible nitric oxide synthase, interleukin-35, myeloid-derived suppressor cell, psoriasis

## Abstract

Although psoriasis is classified as a T cell-mediated inflammatory disease, the contribution of myeloid cells to the pathogenesis of psoriasis is not fully understood. In the present study, we demonstrated that the expression of the anti-inflammatory cytokine interleukin-35 (IL-35) was significantly increased in patients with psoriasis with a marked increase in the number of myeloid-derived suppressor cells (MDSCs). Similar results were obtained in an imiquimod-induced psoriasis mouse model. IL-35 reduced the total number of MDSCs and their subtypes in the spleens and psoriatic skin lesions, ameliorating psoriasis. IL-35 also reduced the expression of inducible nitric oxide synthase in MDSCs, although it had no significant effect on interleukin-10 expression. Adoptive transfer of MDSCs from imiquimod-challenged mice aggravated the disease and weakened the effect of IL-35 in the recipient mice. In addition, mice transferred with MDSCs isolated from inducible nitric oxide synthase knockout mice had milder disease than those with wild-type MDSCs. Furthermore, wild-type MDSCs reversed the effects of IL-35, while MDSCs isolated from inducible nitric oxide synthase knockout mice did not affect IL-35 treatment. In summary, IL-35 may play a critical role in the regulation of iNOS-expressing MDSCs in the pathogenesis of psoriasis, highlighting IL-35 as a novel therapeutic strategy for patients with chronic psoriasis or other cutaneous inflammatory diseases.

## 1 Introduction

Psoriasis is a chronic inflammatory, immune-mediated skin disease caused by vascular hyperplasia, abnormal keratinocyte proliferation, and inflammatory cell infiltration in the epidermis and dermis. Inflammatory cell infiltrates include T lymphocytes, mast cells, macrophages, myeloid-derived suppressor cells (MDSCs), and neutrophils (1). Studies on the pathogenesis of psoriasis have expanded our understanding of skin immunology and facilitated the introduction of innovative and effective therapies. Although it is clear that immune cells and immune-related cytokines are involved in the development of psoriasis, the pathogenesis of psoriasis is still not fully understood.

Interleukin (IL)-35 is an inhibitory cytokine consisting of Epstein-Barr virus-induced gene 3 (EBI3) and IL-12 chain (p35) subunits that belongs to the IL-12 cytokine family (2). IL-35 inhibits of T cell proliferation in various disease models (3, 4) as well as the development and differentiation of T helper 17 (Th17) cells (5, 6). IL-35 plays important roles in many inflammation-related diseases. IL-35 can improve the severity of acute colitis model by promoting the secretion of IL-10 and inhibiting the expression of IL-6, tumor necrosis factor (TNF)-α and IL-17 (7). IL-35 gene therapy alleviated psoriasis-like symptoms in psoriasis mouse models (8). However, the exact mechanisms for IL-35 in the pathogenesis of psoriasis remains incompletely explained.

MDSCs are immature heterogeneous myeloid-derived progenitor cells that can be divided into two subtypes: mononuclear (M-) and polymorphonuclear (also known as granulocytic [G-]) MDSCs (9, 10). MDSCs modulate immune responses in inflammatory bowel disease (11, 12), transplantation (13, 14), many types of cancer (15–17), and infection (18, 19). Recent studies have revealed the expansion of MDSCs, which produce cytokines including IL-23, IL-1β, and C-C motif chemokine ligand 4, in patients with psoriasis (20–23). M-MDSCs express high levels of inducible nitric oxide synthase (iNOS), a mediator of the suppressive function of M-MDSCs in cancer (24). MDSCs function as immune disruptors in patients with systemic lupus erythematosus in an iNOS-dependent manner. The number of M-MDSCs and the expression of iNOS decreased following treatment, suggesting that these cells can be used as an indicator of treatment efficacy (25). In this study, we found that IL-35 significantly inhibited MDSCs in IMQ-induced psoriasis mice. Therefore, we investigated the mechanism of IL-35 regulation of MDSCs in the pathogenesis of psoriasis.

## 2 Materials and methods

### 2.1 Isolation of human peripheral blood mononuclear cells (PBMCs)

All procedures were approved by the Institutional Review Board of Jining Medical University and were conducted in accordance with the 1964 Declaration of Helsinki and its later amendments. All participants provided written, informed consent. Healthy individuals (n = 10) and patients with psoriasis (n = 14) were randomly selected and subjected to peripheral blood drawing (6 mL/person). Human PBMCs were isolated by density-gradient centrifugation using Ficoll-Paque (GE Healthcare Bio-Science AB, Uppsala, Sweden). Plasma samples were stored at –80°C for subsequent analysis. PBMCs at a concentration of 1 × 10^6^/mL were maintained in Roswell Park Memorial Institute 1640 medium (Thermo Fisher Scientific, Waltham, MA, USA) supplemented with 10% fetal bovine serum (Thermo Fisher Scientific).

### 2.2 Imiquimod (IMQ)-induced psoriasis mouse model and treatment

C57BL/6 mice (8 weeks old) were administered a daily dose of 62.5 mg/mouse IMQ cream (Mingxin Pharmaceutical Co. Ltd., Chengdu, China) on their shaved back skin for 7 days and the mice were randomly and blindly divided into control and experimental groups. To determine the therapeutic potential of IL-35, we administered IL-35 (5 μg) (Sino Biological, Beijing, China) one day before the establishment of the IMQ-induced psoriasis mouse model, with three additional injections administered every other day (Days 2, 4, and 6) during the establishment of the model. Twenty-four hours after the last injection, the mice were photographed. The mice were euthanized, and serum and psoriatic lesions were collected. The spleen and skin were used for flow cytometry and histological examination. All animal procedures were approved by the Institutional Animal Ethics Committee (approval number: 2018-JC-003).

### 2.3 Evaluation of psoriasis severity

To evaluate the severity of inflammation in the ears and neck skin of mice, we used an objective scoring system based on the clinical Psoriasis Area and Severity Index (PASI). Erythema, scaling, and thickening were scored independently on a scale of 0 to 4: 0, none; 1, slight; 2, moderate; 3, marked; 4, very marked (26).

### 2.4 Hematoxylin and eosin (H&E) staining and microscopy

Mouse ear and dorsal skin tissues were fixed in 4% paraformaldehyde, embedded in paraffin, sectioned, and stained with H&E. Images were acquired using an Olympus BX600 microscope (Olympus Corp., Tokyo, Japan) and assessed using the Baker scoring system (27).

### 2.5 Enzyme-linked immunosorbent assay (ELISA)

The expression of human C-X-C motif chemokine ligand 8 (CXCL8, Cat: 431507) and IL-6 (Cat: 430507) in the cell culture supernatant and that of other proinflammatory factors, including mouse IL-17A (Cat: 432501), IL-1β (Cat: 432604), and IL-6 (Cat: 431307), in the serum and skin of mice were detected by ELISA purchased from Biolegend (San Diego, CA, USA). Mouse IL-23 ELISA kit (CSB-E-08463m) was provided by CUSABIO (Wuhan, China). The plates were washed with phosphate-buffered saline (PBS) containing Tween-20 (0.05%) and blocked with assay diluent for 1 hour at 37°C. After washing, samples were serially diluted with assay diluent, added to the plates, and incubated for 2 hours at 37°C, followed by the addition of diluted detection antibody at 37°C. The plates were washed again. Avidin-horseradish peroxidase was added and incubated for 30 minutes at 37°C. Tetramethylbenzidine substrate solution was then added and incubated for 15 minutes at room temperature. The reaction was stopped by adding 100 μL of acid stop solution. Absorbance was measured at 450 nm within 20 minutes using an ELISA reader (Bio-Rad Laboratories, RRID : SCR_008426).

### 2.6 Flow cytometry

PBS supplemented with 2% heat-inactivated fetal bovine serum was used as the staining buffer for flow cytometry. PBMCs from healthy individuals and patients with psoriasis were resuspended in staining buffer for extracellular staining of CD4, CD19, CD14, CD15, CD11b, and human leukocyte antigen DR (HLA-DR), as well as intracellular staining of EBI3 and p35. Directly-labelled isotype-matched rat anti-mouse antibodies (BioLegend, San Diego, CA, USA) were used as controls. For intracellular staining, the cells were first stimulated with 50 ng/mL phorbol myristate acetate (Sigma-Aldrich, St. Louis, MO, USA) and 1.0 μg/mL ionomycin (Sigma-Aldrich) for 2 hours and then cultured for 4 hours in the presence of 2 μL monensin (BioLegend). After extracellular staining, the PBMCs were fixed and permeabilized (Fixation/Permeabilization Solution Kit; BD Biosciences, St. Louis, MO, USA), according to the manufacturer’s protocol. EBI3 and p35 staining were subsequently performed.

The spleens of treated mice were collected, homogenized, and suspended as single cells in red blood cell lysis buffer. Skin tissues from treated mice were collected, added with type IV collagenase (2 mg/ml, Cat: 17104019, Gibco) and DNase I (25 U/ml, Cat: SSNP0, SinoBiological) in 1640 medium, with shock digestion for 2 hours at 220 r/min at 37 °C. After filtration and centrifugation, bottom cells were suspended as single cells flow cytometry. We used Zombie Aqua™ (Cat: 423101, Biolegend) for dead cell exclusion as follows: Single-cell suspension was prepared and cells were washed with PBS buffer (no Tris buffer and protein free). Zombie Aqua™ dye was diluted at 1:500 in PBS. Cells at 1×10^7^ were diluted in 100 µl Zombie Aqua™ solution. Then cells were extracellularly stained with CD11b, Gr-1, Ly6G, and Ly6C for 30 minutes at 4°C in the dark. Directly-labelled, isotype-matched rat anti-mouse antibodies (BioLegend) were used as controls. After washing twice with PBS, the cells were resuspended in 200 mL of PBS and analyzed *via* flow cytometry. For intracellular staining, the cells were stained extracellularly and then fixed and permeabilized (Fixation/Permeabilization Solution Kit; BD Biosciences, Franklin Lakes, NJ, USA) for 30 minutes at 4°C. Finally, the cells were intracellularly stained with anti-iNOS and anti-IL-10.

The following antibodies were used: Fluorescein isothiocyanate (FITC) anti-human CD4, anti-mouse CD11b (BioLegend), anti-human HLA-DR (BD Biosciences). Brilliant Violet 421 (BV421) anti-human CD19, anti-human CD11b (BioLegend). phycoerythrin (PE) anti-human CD15 (BD Biosciences, San Diego, CA, USA), anti-human p35, anti-mouse Ly6G, anti-mouse iNOS, anti-mouse IL-10 (BioLegend). Allophycocyanin (APC) anti-human EBI3, anti-mouse Gr-1, anti-mouse Ly6C (BioLegend).

### 2.7 *In vitro* preparation of bone marrow-derived MDSCs

Bone marrow cells were isolated from mice by flushing their femurs and tibiae, centrifuging the elution at 1,800 rpm for 5 minutes, and resuspending the cells in complete Dulbecco’s modified Eagle medium (DMEM, Invitrogen, Shanghai, China) supplemented with 40 ng/mL IL-6 (Peprotech, Suzhou, China) and 40 ng/mL granulocyte-macrophage colony-stimulating factor (Peprotech). Bone marrow cells were maintained at 37°C in a 5% carbon dioxide humidified atmosphere for 4 days. To investigate the effect of IL-35 on the expansion of MDSCs, IL-35, IL-6, and granulocyte-macrophage colony-stimulating factor were added to the medium simultaneously.

### 2.8 Immunofluorescence staining

We performed immunofluorescence staining for IL-35 and M-MDSCs in the skin of patients with psoriasis and healthy controls and for MDSCs and iNOS^+^ MDSCs in murine skin lesions. Mice were treated with IL-35; the back skin was obtained the day after the last treatment, frozen, and then stained. Samples were incubated with primary antibodies (rat anti-human p35 and CD14, anti-mouse CD11b and iNOS, rabbit anti-human EBI3^+^ and HLA-DR, anti-mouse Gr-1) (Abcam) at 4°C overnight, followed by directly-labelled IgG anti-rat or rabbit antibodies (ZsBio, Beijing, China) for 1 hour. Tissue sections were examined under a fluorescent microscope (Olympus Optical, Tokyo, Japan). Images were captured at zoom magnification.

### 2.9 Adoptive transfer of MDSCs

For adoptive transfer, 2 × 10^6^ CD11b^+^Gr1^+^ cells isolated from the spleen of IMQ-induced (wild-type [WT] and iNOS knockout [iNOS^-/-^]) mice by flow cytometry were washed twice, resuspended in 200 μL PBS and injected into the tail vein. MDSCs were injected the day before the application of IMQ and injected again on the third day after the application of IMQ.

### 2.10 Statistical analysis

All the results were analyzed using the GraphPad Prism (GraphPad Prism, RRID : SCR_002798). Data are presented as means ± standard error (SEM). One-way analysis of variance (ANOVA) and Student’s t-test were used to analyze the differences between multiple groups and two groups, respectively. Statistical significance was set at *P* < 0.05.

## 3 Results

### 3.1 IL-35 expression was significantly increased in patients with psoriasis

**Figure 1:**
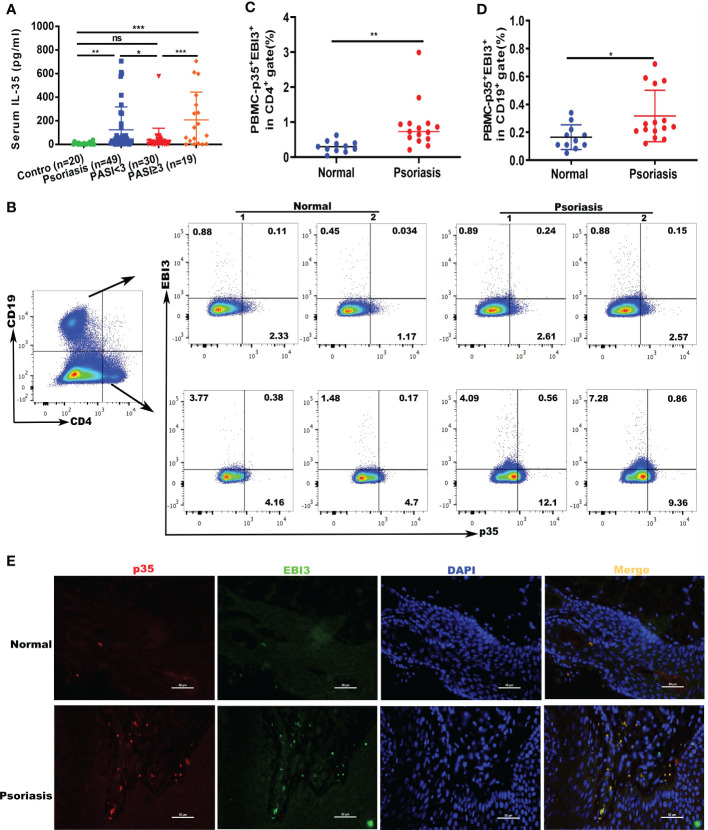
Expression of IL-35 increased in psoriatic serum and peripheral blood specimens. **(A)** Levels of expression of IL-35 in the serum of patients with psoriasis (n=49). Psoriasis Area and Severity Index (PASI) <3 (n=19), PASI ≥3 (n=19), or control (n=20) were measured using ELISA. Values obtained from individual controls and patients are plotted as dots. **(B)** The number of CD4^+^EBI3^+^p35^+^ and CD19^+^EBI3^+^p35^+^ cells in the peripheral blood of patients with psoriasis was detected using flow cytometry. **(C, D)** Flow cytometry proportional analysis results in **(B)**. **(E)** Immunofluorescence staining of IL-35 (p35^+^EBI3^+^) in patients (n=7) and control (n=5) skin lesions. Red represents anti-p35 Ab, green represents anti-EBI3 Ab, yellow represents p35 and EBI3 merged, and blue represents 4′,6-diamidino-2-phenylindole (DAPI). Original magnification 400×. *P<0.05, **P<0.01, ***P<0.001; ns, not significant.

To investigate the involvement of IL-35 in skin diseases, we examined IL-35 levels in the serum of healthy individuals and patients with psoriasis. We recruited patients (n = 53) and age- and sex-matched healthy controls (n = 20); the demographic characteristics are summarized in **Table 1**. ELISA showed that serum IL-35 levels were significantly increased in patients with psoriasis compared to those in healthy controls (P = 0.0089; **Figure 1A**). Moreover, as the symptoms of psoriasis worsened (higher PASI score), the expression of IL-35 increased. Specifically, the expression of IL-35 in specimens with a PASI score ≥ 3 was significantly higher than that in those with a PASI score < 3 (**Figure 1A**). Flow cytometry also revealed increased populations of CD4^+^EBI3^+^p35^+^ and CD19^+^EBI3^+^p35^+^ cells in the peripheral blood of patients with psoriasis (**Figures 1B–D**). We demonstrated that IL-35 secreted by both T and B lymphocytes was significantly increased (P < 0.01 and P < 0.05, respectively). Moreover, immunofluorescence staining showed that IL-35 expression was significantly increased in the skin of patients with psoriasis (**Figure 1E**).

**Table 1:** Characteristics of Psoriasis patients and Healthy controls for detection of IL-35 in plasma.

Characteristics	Psoriasis patients	Healthy controls
Number of analyzed patients	53	20
Age in years, mean ± SD	36.08 ± 2.276	34.67 ± 2.855
Gender	Males (29), Females (24)	Males (9), Females (11)
Race/ethnicity	Chinese	Chinese
Treatments	None	N/A
PASI score, mean (range) ± SD	4.973 (0.2-32.1) ± 1.155	N/A

N/A, not applicable; PASI, Psoriasis Area and Severity Index.

### 3.2 M-MDSCs were expanded in patients with psoriasis

**Figure 2:**
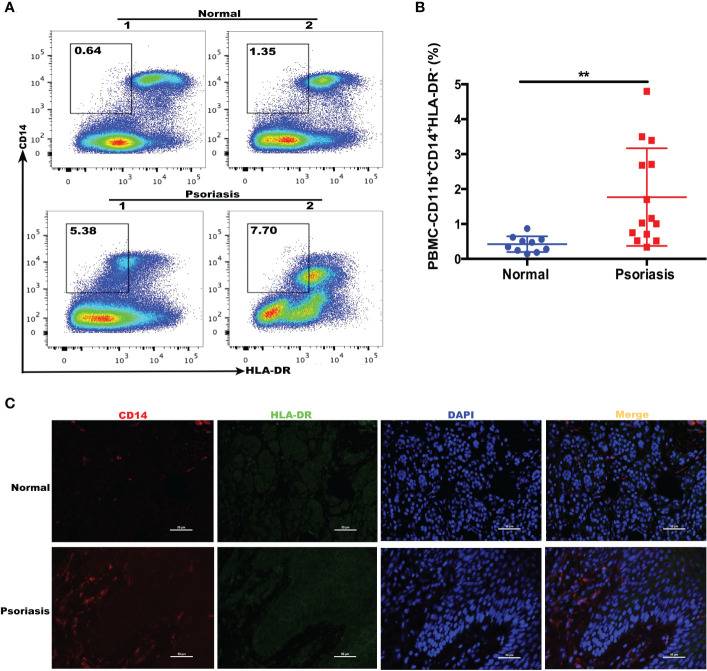
Expansion of MDSCs in the blood of patients with psoriasis. **(A)** HLA-DR and CD14 expression in freshly isolated PBMCs from healthy individuals (controls) and patients with psoriasis (psoriasis). CD11b^+^CD14^+^HLA-DR^-/low^ cells were gated (shown by a small window). **(B)** Scatter plots of M-MDSCs from PBMCs. **(C)** Immunofluorescence staining of M-MDSCs (CD14^+^HLA-DR^-^) in skin lesions in patients with psoriasis (N = 7) and controls (N = 5). Red represents anti-CD14; green, anti-HLA-DR; yellow, CD14 and HLA-DR merged; and blue, DAPI. Original magnification, 400×. **P < 0.01. DAPI, 4’,6-diamidino-2-phenylindole; HLA-DR, human leukocyte antigen DR; MDSC, myeloid-derived suppressor cell; M-MDSC, mononuclear MDSC; PBMC, peripheral blood mononuclear cell.

We examined whether the number of M-MDSCs was altered in patients with psoriasis. We recruited patients (n = 14) and age and sex-matched healthy donors (n = 10); the demographics are summarized in **Table 2**. Flow cytometry confirmed that the number of human M-MDSCs (CD11b^+^CD14^+^HLA-DR^-^) was considerably higher in the peripheral blood of patients with psoriasis (*P* = 0.0065; **Figures 2A, B**). Immunofluorescence staining of CD14^+^HLA-DR^-^ M-MDSCs in the inflamed skin of patients with psoriasis revealed that the infiltration of M-MDSCs in psoriatic skin tissue increased significantly compared to that in healthy controls (**Figure 2C**).

**Table 2:** Characteristics of Psoriasis patients and Healthy controls for detection of MDSCs.

Characteristics	Psoriasis	Healthy
Number of analyzed patients	14	10
Age in years, mean ± SD	34.6 ± 5.221	37.6 ± 3.036
Gender	Males (6), Females (8)	Males (4), Females (6)
Race/ethnicity	Chinese	Chinese
Treatments	None	N/A
PASI score, mean (range) ± SD	2 (1.5-3) ± 3.609	N/A

N/A, not applicable; PASI, Psoriasis Area and Severity Index.

### 3.3 MDSCs populations increased in mice with IMQ-induced psoriasis

**Figure 3:**
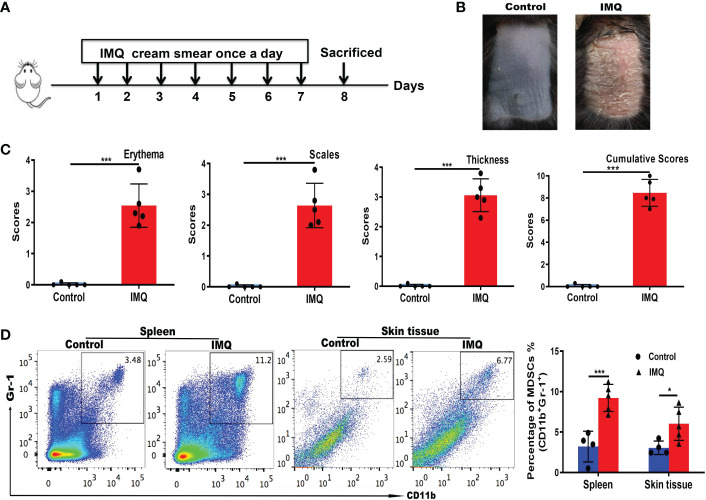
MDSC populations increased in mice with IMQ-induced psoriasis. **(A)** Experimental procedures and establishment of an IMQ-induced psoriasis mouse model. **(B)** Murine back skin specimens. **(C)** PASI scoring of erythema, scaling, and thickness, and the cumulative scores for different groups. **(D)** Detection of MDSCs in the spleen and skin by flow cytometry and subsequent quantification. *P < 0.05, ***P < 0.001. IMQ, imiquimod; MDSC, myeloid-derived suppressor cell; PASI, Psoriasis Area and Severity Index.

The IMQ-induced psoriasis mouse model is the most common model of psoriasis (28). The experimental procedures and acquisition of images of mouse back skin are shown in **Figures 3A, B**. Based on PASI scoring of erythema, scaling, and thickness, we determined the cumulative scores for different groups (**Figure 3C**). The IMQ group had higher cumulative scores than the control group. Flow cytometry was used to detect changes in MDSCs populations in the spleen and skin of mice with IMQ-induced psoriasis. The numbers of MDSCs in the spleen and skin of mice in the IMQ group were significantly higher than those in the control group (*P* < 0.001 and *P* < 0.05, respectively; **Figure 3D**). The results were consistent with those obtained using clinical samples.

### 3.4 IL-35 treatment relieved the symptoms of psoriasis in models

We stimulated HaCaT cells with M5 in combination with IL-35 to observe the effect of IL-35 on CXCL8 and IL-6 expression. We found consistently lower levels of CXCL8 and IL-6 in the IL-35-treated group than in the control group, suggesting that IL-35 suppresses the expression of CXCL8 and IL-6 in stimulated HaCaT cells (**Figures S1A, B**).

**Figure 4:**
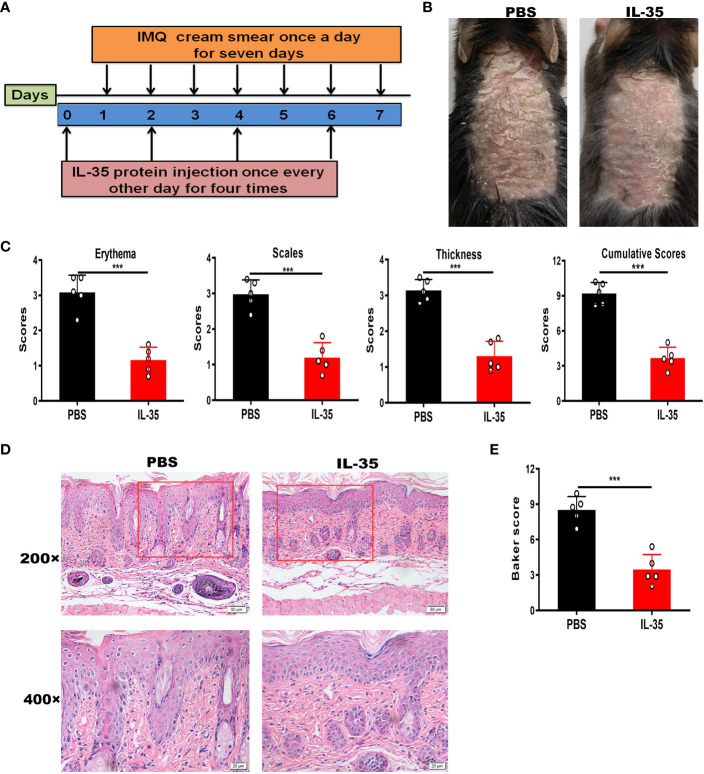
IL-35 treatment reduces the inflammatory process in mice with IMQ-induced psoriasis. **(A)** Schedule of IL-35 administration. **(B)** Skin phenotype after IL-35 treatment (N = 6). **(C)** PASI scoring of erythema, scaling, and thickness, and the cumulative scores for different groups. **(D)** H&E-stained skin sections (200× and 400× magnification; the 400× image is the enlarged image in the box). **(E)** Pathological scores of skin sections using the Baker scoring system. Data are presented as means ± standard deviation from three independent experiments. ***P < 0.001. H&E, hematoxylin and eosin; IL, interleukin; IMQ, imiquimod; PASI, Psoriasis Area and Severity Index.

Next, we determined whether IL-35 has a therapeutic effect in the IMQ-induced psoriasis mouse model. The schedule of IL-35 administration in mice with IMQ-induced psoriasis is shown in **Figure 4A**. After treatment, the control group had serious inflammation and skin flaking, which had worsened by the end of the experiment (**Figure 4B**). Based on PASI scoring of erythema, scaling, and thickness (**Figure 4C**), we determined the cumulative scores for different groups (**Figure 4C**). The IL-35 group had lower cumulative scores than the control group. H&E staining revealed reduced epidermal thickening in the IL-35 group compared to the control group (**Figure 4D**). The Baker score of the IL-35-treated group was significantly lower than that of the control group (*P* < 0.001; **Figure 4E**).

To further investigate the therapeutic effect of IL-35, we treated IMQ-induced psoriasis mice with p35 neutralizing antibodies. The symptoms in the p35 neutralizing antibody group were not only substantially worse than those in the IL-35-treated group but also worse than those in the IgG control group (**Figure S2**).

We also explored whether IL-35 could have a therapeutic effect on K14-VEGF-A-Tg mice. K14-VEGF-A-Tg mice (10 weeks old) were injected with 5 μg IL-35 recombinant protein (**Figure S3A**). Mice in the control group showed increased ear thickening; however, mice in the IL-35-treated group were healthy (**Figure S3B**). Pathological features, such as erythema, scaling, and thickness, based on PASI scores, are shown in **Figure S3C**. The IL-35-treated group had lower cumulative scores than the control group (**Figure S3C**). H&E staining revealed the severity of the disease in the control group (**Figure S3D**; left panel). Conversely, a reversal in ear thickness and reduction in leukocyte infiltration were observed in the dermis and epidermis of the IL-35-treated group (**Figure S3D**; right panel). Moreover, the IL-35-treated group had a lower disease score, based on the Baker scoring system, than the control group (**Figure S3E**).

### 3.5 IL-35 treatment reduced the number of recruited MDSCs

**Figure 5:**
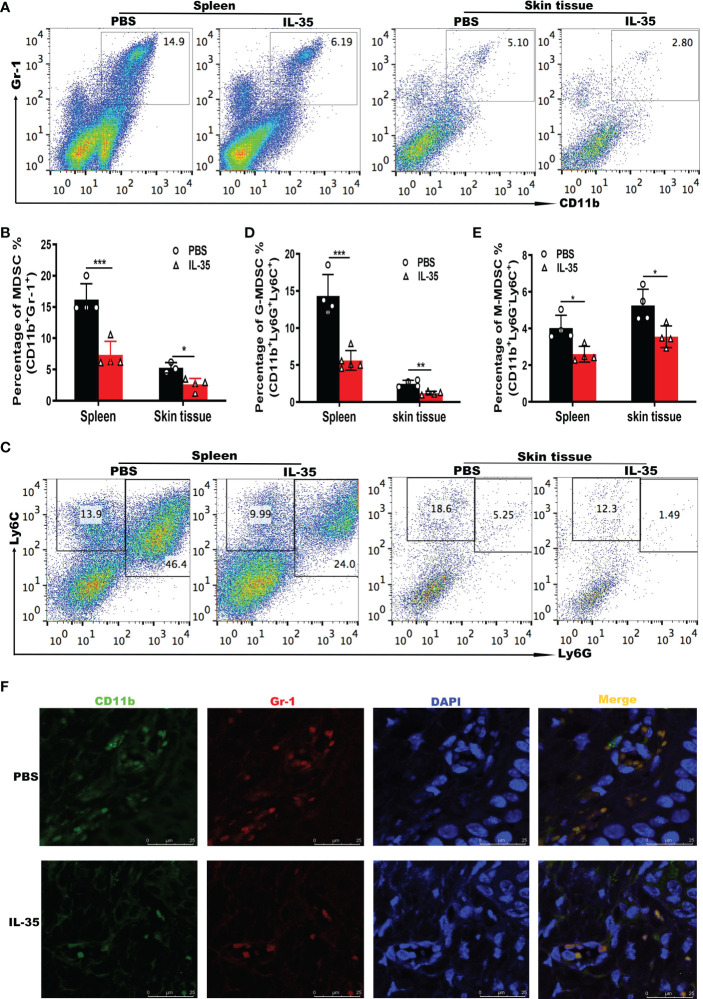
IL-35 suppresses MDSCs infiltration in mice with IMQ-induced psoriasis.**(A)** Representative fluorescence-activated cell sorting plots of MDSCs (CD11b^+^Gr-1^+^) in the spleen and skin (gated on CD45) 24 hours after the last injection of IL-35. **(B)** Flow cytometry statistics of the results in **(A)**. **(C)** Representative fluorescence-activated cell sorting plots of G-MDSCs (CD11b^+^Ly6G^+^Ly6C^low^) and M-MDSCs (CD11b^+^Ly6G^-^Ly6C^high^) in the spleen and skin (gated on CD45). **(D–E)** Quantification of flow cytometry results in **(C)**. **(F)** Immunofluorescence staining of infiltrated CD11b^+^Gr-1^+^ MDSCs in murine skin lesions (N = 6). Green represents anti-CD11b; red, anti-Gr-1; yellow, CD11b and Gr-1 merged; and blue, DAPI. Original magnification, 400×. Data are presented as means ± standard deviation. *P < 0.05, **P < 0.01, ***P < 0.001. DAPI, 4’,6-diamidino-2-phenylindole; G-MDSC, granulocyte MDSC; IL, interleukin; IMQ, imiquimod; M-MDSC, mononuclear MDSC; MDSC, myeloid-derived suppressor cell.

The above results showed that IL-35 had a therapeutic effect on psoriasis, and MDSCs were expanded in patients with psoriasis and in the mouse model. To test whether IL-35 ameliorates psoriasis by reducing the accumulation of MDSCs, we used flow cytometry to analyze the number of MDSCs in mice with IMQ-induced psoriasis treated with or without IL-35. Compared with that in the control group, the administration of IL-35 reduced the infiltration of MDSCs in the spleen and skin of mice with IMQ-induced psoriasis (**Figures 5A, B**).

Next, we investigated whether G-MDSCs and M-MDSCs populations were altered in response to IL-35 treatment. In the spleen and skin, both G-MDSCs and M-MDSCs populations were substantially reduced (**Figures 5C–E**). Immunofluorescence staining of CD11b^+^Gr-1^+^ MDSCs in inflamed skin showed that IL-35 treatment inhibited the accumulation of MDSCs (**Figure 5F**). These results demonstrated that the immunosuppressive effect of IL-35 on IMQ-induced psoriasis was achieved by reducing the infiltration and proportion of MDSCs. IL-35 can also alter the proportions of MDSCs subtypes. Finally, we examined whether IL-35 directly affects the differentiation and recruitment of MDSCs *in vitro* using fluorescence-activated cell sorting. IL-35 had little effect on the differentiation of MDSCs (**Figures S4A, B**). IL-35 also did not significantly affect the numbers of G-MDSCs and M-MDSCs (**Figures S4C–E**).

### 3.6 IL-35 regulates inflammatory cytokine production in the systemic and local immune microenvironment in IMQ-induced mice

**Figure 6:**
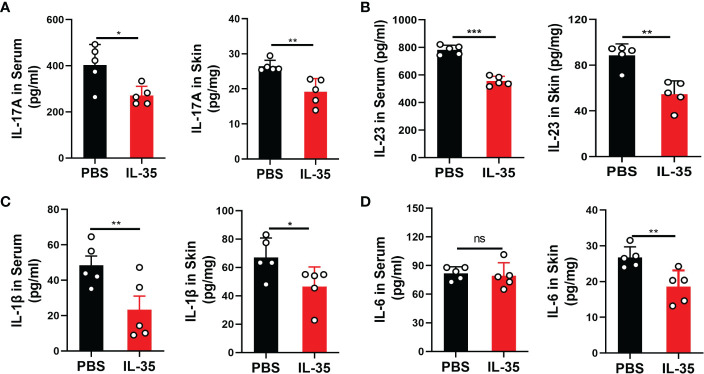
IL-35 inhibited the secretion of inflammatory cytokines in the serum and local lesions of mice with IMQ-induced psoriasis. Several proinflammatory cytokines were detected by ELISA in serum and skin tissue after IL-35 treatment. Cytokines in supernatants of extracted tissue protein were assayed and are presented as picograms of cytokine per milligram of tissue. **(A)** IL-17A, **(B)** IL-23, **(C)** IL-1β, and **(D)** IL-6. Data are presented as means ± standard deviation from three independent experiments. *P < 0.05, **P < 0.01, ***P < 0.001. ELISA, enzyme-linked immunosorbent assay; IL, interleukin; IMQ, imiquimod; ns, not significant.

Inflammatory cytokines play an important role in the progression of psoriasis (29). ELISA was used to measure cytokine levels in serum and dorsal skin. Cytokines in supernatants of extracted tissue protein were assayed and are presented as picograms of cytokine per milligram of tissue. IL-17A (**Figure 6A**), IL-23 (**Figure 6B**), IL-1β (**Figure 6C**), and IL-6 (**Figure 6D**) were detected in serum and skin tissue. These results showed that, although there was no significant difference in serum IL-6 levels, other cytokines were significantly different in serum and skin tissue.

### 3.7 Adoptive transfer of MDSCs weakened the effect of IL-35 in mice with IMQ-induced psoriasis

**Figure 7:**
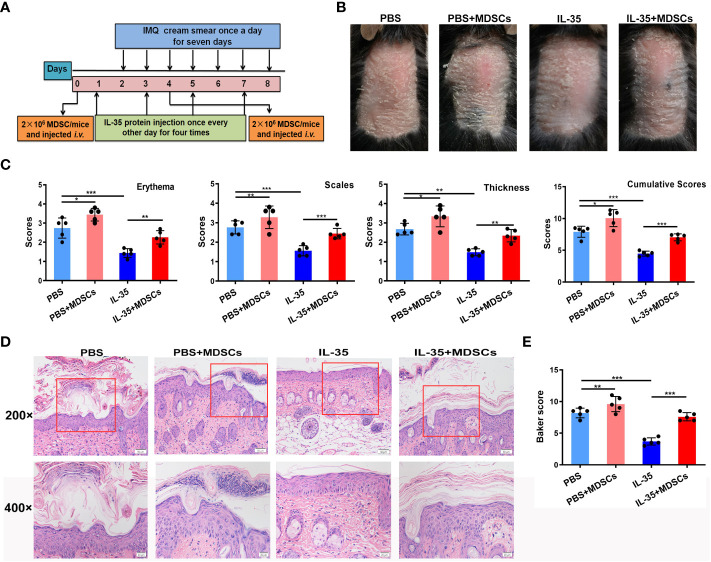
Adoptive transfer of MDSCs weakens the effect of IL-35 in mice with IMQ-induced psoriasis. **(A)** Experimental schedule. **(B)** Representative phenotype of murine skin after adoptive transfer of MDSCs. **(C)** PASI scoring of erythema, scaling, and thickness, and the cumulative scores for different groups. **(D)** H&E-stained skin sections (200× and 400× magnification; the 400× image is the enlarged image in the box). **(E)** Pathological scores of skin sections (N = 6) using the Baker scoring system. Data are presented as means ± standard deviation. *P < 0.05, **P < 0.01, ***P < 0.001. H&E, hematoxylin and eosin; IL, interleukin; IMQ, imiquimod; MDSC, myeloid-derived suppressor cell; PASI, Psoriasis Area and Severity Index.

To further explore whether the therapeutic effect of IL-35 was related to MDSCs, MDSCs from IMQ-induced mice were adoptively transferred, following the experimental schedule shown in **Figure 7A**. We found that adoptive transfer of MDSCs from IMQ-induced mice aggravated disease progression. When MDSCs from IMQ-induced mice were transferred to mice in the IL-35-treated group, psoriasis symptoms worsened in these mice compared to those treated with IL-35 alone (**Figure 7B**). We evaluated pathological features, such as erythema, scaling, and thickness, using the PASI scoring system, and found that, compared to the control group, the MDSCs group had higher PASI scores (**Figure 7C**). Similarly, the IL-35+MDSCs group had higher PASI scores than the IL-35-treated group. We determined the cumulative scores for different groups and found that the IL-35-treated group had the lowest cumulative scores (**Figure 7C**).

H&E staining revealed increased epidermal thickening in the MDSCs group compared to the control group. The epidermis in the IL-35+MDSCs group was substantially thicker than that in the IL-35-treated group (**Figure 7D**). Moreover, the Baker score of the IL-35-treated group was the lowest of the four groups (**Figure 7E**).

### 3.8 IL-35 inhibited iNOS expression in MDSCs in mice with IMQ-induced psoriasis

**Figure 8:**
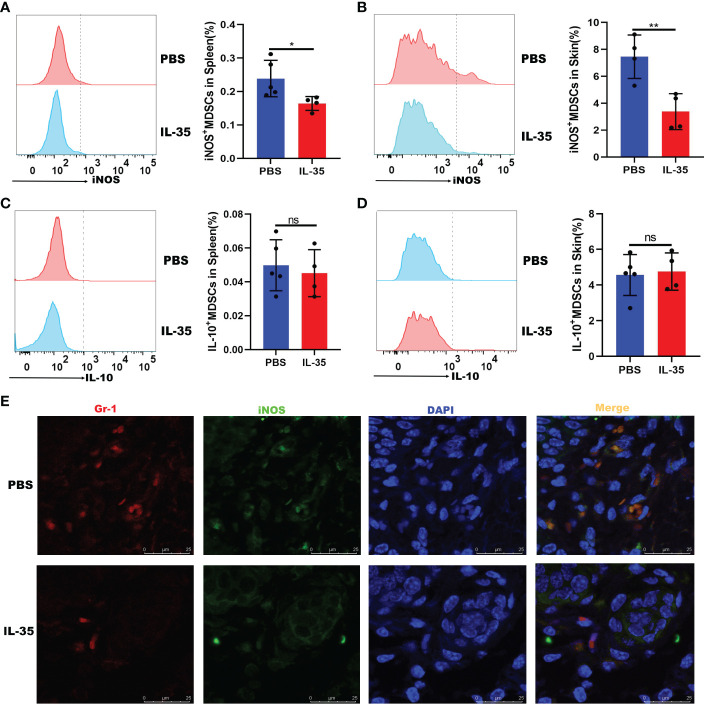
IL-35 inhibited the expression of iNOS in MDSCs. IL-35 recombinant protein was used to treat IMQ-induced psoriasis in a mouse model. The expression of iNOS and IL-10 was evaluated. Fluorescence-activated cell sorting analysis of iNOS and IL-10 expression in **(A)** and **(C)** the spleen (gated on CD11b^+^Gr-1^+^) and **(B)** and **(D)** the skin (gated on CD45^+^CD11b^+^Gr-1^+^). **(E)** Immunofluorescence staining of infiltrated iNOS^+^Gr-1^+^ MDSCs in skin lesions (N = 5). Green represents anti-iNOS; red, anti-Gr-1; yellow, iNOS and Gr-1 merged; and blue, DAPI. Original magnification, 400×. Data are presented as means ± standard deviation. *P < 0.05, **P < 0.01. DAPI, 4’,6-diamidino-2-phenylindole; IL, interleukin; IMQ, imiquimod; iNOS, inducible nitric oxide synthase; MDSC, myeloid-derived suppressor cell; ns, not significant.

The above results indicated that IL-35 inhibits the recruitment of MDSCs. Next, we determined whether IL-35 affects the expression of inflammatory mediators in MDSCs. Flow cytometry was used to analyze the expression of iNOS and IL-10, which were secreted by MDSCs in mice with IMQ-induced psoriasis treated with or without IL-35. The results showed that, compared to the control group, the IL-35-treated group had reduced iNOS expression in the spleen and skin (**Figures 8A, B**). However, there was no difference in the level of IL-10 (**Figures 8C, D**). Immunofluorescence staining of iNOS^+^Gr-1^+^ MDSCs in inflamed skin showed that iNOS^+^Gr-1^+^ MDSCs accumulation was inhibited by IL-35 (**Figure 8E**).

### 3.9 Adoptive transfer of iNOS^-/-^ MDSCs had no effect on the function of IL-35 in mice with IMQ-induced psoriasis

**Figure 9:**
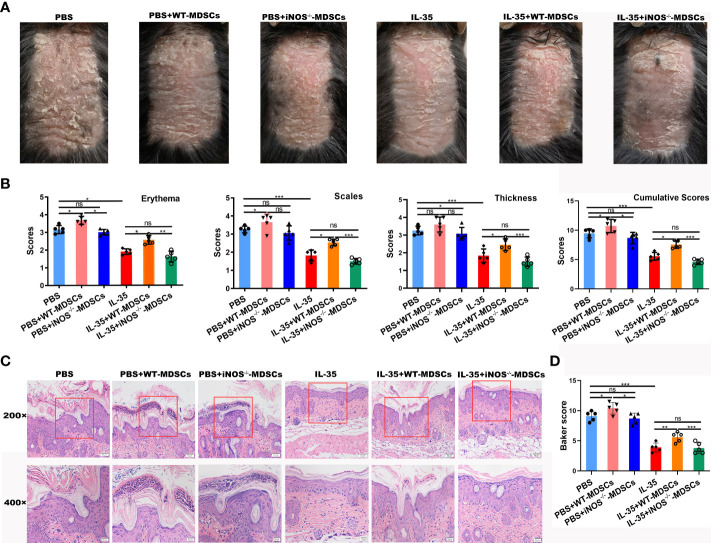
Adoptive transfer of iNOS^-/-^ MDSCs had no effect on the function of IL-35 in mice with IMQ-induced psoriasis. **(A)** Representative phenotype of murine skin after adoptive transfer of WT-MDSCs and iNOS^-/-^ MDSCs. **(B)** PASI scoring of erythema, scaling, and thickness, and the cumulative scores for different groups. **(C)** H&E-stained skin sections (200× and 400× magnification; the 400× image is the enlarged image in the box). **(D)** Pathological scores of skin sections (N = 5) using the Baker scoring system. Data are presented as means ± standard deviation. *P < 0.05, **P < 0.01, ***P < 0.001. H&E, hematoxylin and eosin; IL, interleukin; IMQ, imiquimod; iNOS, inducible nitric oxide synthase; MDSC, myeloid-derived suppressor cell; ns, not significant; PASI, Psoriasis Area and Severity Index; WT, wild-type.

To explore the role of iNOS^+^ MDSCs in the pathogenesis of psoriasis, MDSCs from WT or iNOS^-/-^ IMQ-induced mice were adoptively transferred, following the experimental schedule shown in **Figure 7A**. The adoptive transfer of WT-MDSCs derived from mice with IMQ-induced psoriasis aggravated disease progression in the PBS group (PBS+WT-MDSCs). However, there was no exacerbation of symptoms when MDSCs from IMQ-induced iNOS^-/-^ mice were transferred to the PBS group (PBS+iNOS^-/-^ MDSCs; **Figure 9A**). Moreover, when WT-MDSCs were transferred to the IL-35-treated group, psoriasis symptoms were aggravated in these mice compared to those treated with IL-35 alone. There was no significant difference between the IL-35+iNOS^-/-^ MDSCs and IL-35-treated groups (**Figure 9A**). We evaluated pathological features, such as erythema, scaling, and thickness, using the PASI scoring system, and found that, compared to the PBS group, the PBS+WT-MDSCs group had higher PASI scores (**Figure 9B**). Similarly, the IL-35+WT-MDSCs group had higher PASI scores than the IL-35-treated group. The PBS+iNOS^-/-^ MDSCs group had similar PASI scores to those of the PBS group. There was no significant difference in PASI scores between the IL-35+iNOS^-/-^ MDSCs and IL-35-treated groups. We determined the cumulative scores for different groups and found that the cumulative scores were comparable between the PBS+iNOS^-/-^ MDSCs group and the PBS group, and between the IL-35-treated group and the IL-35+iNOS^-/-^ MDSCs group (**Figure 9B**).

H&E staining revealed increased epidermal thickening, severe hyperkeratosis, and incomplete keratosis in the PBS+WT-MDSCs group compared to the control group. The PBS+iNOS^-/-^ MDSCs group had reduced hyperkeratosis and incomplete keratosis compared to the PBS+WT-MDSCs group but similar morphology to that of the PBS group. The epidermis in the IL-35+WT-MDSCs group was substantially thicker than that in the IL-35-treated group; however, the IL-35+iNOS^-/-^ MDSCs group had similar morphology to that of the IL-35-treated group (**Figure 9C**). Moreover, the Baker score of the IL-35-treated group was the lowest of the six groups (**Figure 9D**). The Baker scores were consistent with the phenotype of each group.

## 4 Discussion

Psoriasis, a common chronic inflammatory skin disease, is primarily mediated by the pathological crosstalk between immune cells and epidermal keratinocytes (30), which includes infiltrating T cells, macrophages, dendritic cells, MDSCs, and neutrophils (31–33). The IL-23-IL-17A-Th17 axis also plays a critical role in the development of psoriasis (34, 35).

IL-35 plays a critical role in several immune-related diseases, such as autoimmune diseases, viral and bacterial infections, and tumors. Wirtz et al. (3) reported that enteritis symptoms in an inflammatory bowel disease mouse model were significantly reduced following vector-mediated overexpression of IL-35. However, few studies have explored the role of IL-35 in the pathogenesis of psoriasis. Li et al. (36) revealed that serum IL-35 levels were higher in patients with psoriatic arthritis than in patients with psoriasis and healthy controls. Cardoso et al. (37) reported no differences in serum IL-35 levels between Brazilian patients with psoriasis and healthy controls. Wei et al. (38) showed that the expression of IL-35 in the peripheral blood of patients with psoriasis vulgaris was lower than that in controls. Similarly, Deng et al. (39) showed that plasma IL-35 levels were lower in patients with psoriasis than in healthy individuals. However, Placek et al. (40) showed that serum IL-35 levels were higher in patients with psoriasis but without any statistically significant relationship with PASI. These studies detected the expression of IL-35 in patients with psoriasis; however, few studies have determined the specific role of IL-35 in psoriasis. In this study, we detected a significant increase in serum IL-35 levels in patients with psoriasis. The number of EBI3^+^p35^+^ cells was also significantly increased in the peripheral blood of patients with psoriasis (**Figure 1**). As an anti-inflammatory cytokine, IL-35 expression was significantly increased in patients with psoriasis, possibly to combat the severe inflammatory response to psoriasis.

We previously revealed that the overexpression of IL-35 inhibited the expression of proinflammatory factors in an *in vitro* model of psoriasis and ameliorated the disease indexes of psoriasis model mice (8). Similarly, the administration of IL-35 recombinant protein improved the severity of psoriasis in mice; however, the specific mechanism of IL-35 recombinant protein was not explored further (7). We simultaneously treated IMQ-induced psoriasis mice with p35 neutralizing antibodies (a subunit of IL-35). The results showed that p35 neutralizing antibodies aggravated the symptoms of psoriasis in mice. P35 was not only associated with EBI but also with p40, forming another heterodimeric cytokine, IL-12. Anti-p35 monoclonal antibodies blocked the effects of both IL-35 and IL-12. IL-12p40 inhibition could be a potential treatment for psoriasis (41, 42). These experiments suggest that the exacerbation of symptoms in psoriatic mice, caused by p35 neutralizing antibodies, is due to the blocking of IL-35.

We previously reported that IL-35 gene therapy exerts immunosuppressive functions by inhibiting macrophage recruitment and regulating the ratio of M1/M2, thereby improving the pathogenesis of psoriatic mice. In addition, we found that IL-35 expression inhibited the proportion of CD11b^+^ myeloid cells, suggesting that IL-35 may regulate myeloid cells (8). Therefore, we focused on MDSCs. In healthy individuals, immature myeloid cells (IMCs) generated in bone marrow quickly differentiate into mature granulocytes, macrophages or dendritic cells (DCs). In pathological conditions such as cancer, various infectious diseases, sepsis, trauma, bone marrow transplantation or some autoimmune disorders, a partial block in the differentiation of IMCs into mature myeloid cells results in an expansion of this population. Importantly, the activation of these cells in a pathological context results in the upregulated expression of immune suppressive factors such as arginase (encoded by Arg1) and inducible nitric oxide synthase (iNOS; also known as NOS2) and an increase in the production of NO (nitric oxide) and reactive oxygen species (ROS). Together, this results in the expansion of an IMC population that has immune suppressive activity, these cells are collectively known as MDSCs (43). MDSCs were originally identified by their CD11b^+^Gr-1^+^ phenotype in tumor-bearing mice (44). However, as the Gr-1 gene homolog is lacking in humans, the surface markers of human MDSCs are different from those of mice (e.g., CD14^+^HLA-DR^-/low^) (45). Cao et al. (20) reported an increase in the number of M-MDSCs in patients with psoriasis, and further studied the abnormal effects on their regulated function. Other studies revealed a marked increase in the number of MDSCs in psoriasis; however, these cells also lack sufficient immunosuppressive functions (33, 46, 47). Peng et al. (23) reported that MDSCs play a proinflammatory role in IMQ-induced psoriasis-like skin inflammation, by regulating the infiltration of CD4^+^ T cells. The depletion of MDSCs by gemcitabine significantly suppressed the IMQ-mediated psoriatic phenotype, suggesting that targeting MDSCs may serve as a novel strategy for the treatment of psoriasis. Consistent with other studies, we also found an increased number of peripheral blood CD11b^+^CD14^+^HLA-DR**^−^** ^/low^ MDSCs in patients with psoriasis compared to healthy controls. Concomitantly, we also detected a significant increase in the number of MDSCs in the spleen and skin lesions of mice with IMQ-induced psoriasis.

In this study, a marked increase in the number of MDSCs was observed in IMQ-induced psoriatic mice. In contrast, the number of MDSCs was reduced in IMQ-induced psoriatic mice treated with IL-35 recombinant protein. Furthermore, G-MDSCs and M-MDSCs counts in the spleen and skin were reduced in the IL-35-treated group compared to the control group. Despite a substantial reduction in MDSCs in IL-35-treated mice with IMQ-induced psoriasis, IL-35 did not play a direct role in the differentiation of MDSCs. This suggests that MDSCs differentiation is potentially due to the regulation of the immune response in mice.

MDSCs play a deleterious role in cancer progression and infectious diseases; however, their role in autoimmune diseases appears to be more complex (48). To determine whether MDSCs promote or inhibit inflammation, we conducted adoptive transfer experiments. We found that the adoptive transfer of MDSCs weakened the effect of IL-35, consistent with the results of Cao et al. (20). The levels of iNOS and IL-10 were markedly increased in tumor-induced MDSCs, indicating their immunosuppressive function in cancer (49, 50). However, few studies have examined the role of iNOS and IL-10 secreted by MDSCs in autoimmune diseases. Herein, we found that the population of MDSCs secreting iNOS, but not IL-10, was significantly increased in the IMQ-induced mouse psoriasis model. Furthermore, the adoptive transfer of MDSCs from IMQ-induced mice weakened the anti-inflammatory effects of IL-35 in IMQ-induced psoriasis; however, this was reversed by MDSCs from IMQ-induced iNOS^-/-^ mice. We speculate that the increased number of MDSCs does not necessarily have an immunosuppressive function in the IMQ-induced psoriasis mouse model. It is plausible that the immunosuppressive function of MDSCs may be impaired by secreting iNOS, thereby promoting immune responses.

## 5 Conclusions

**Figure 10:**
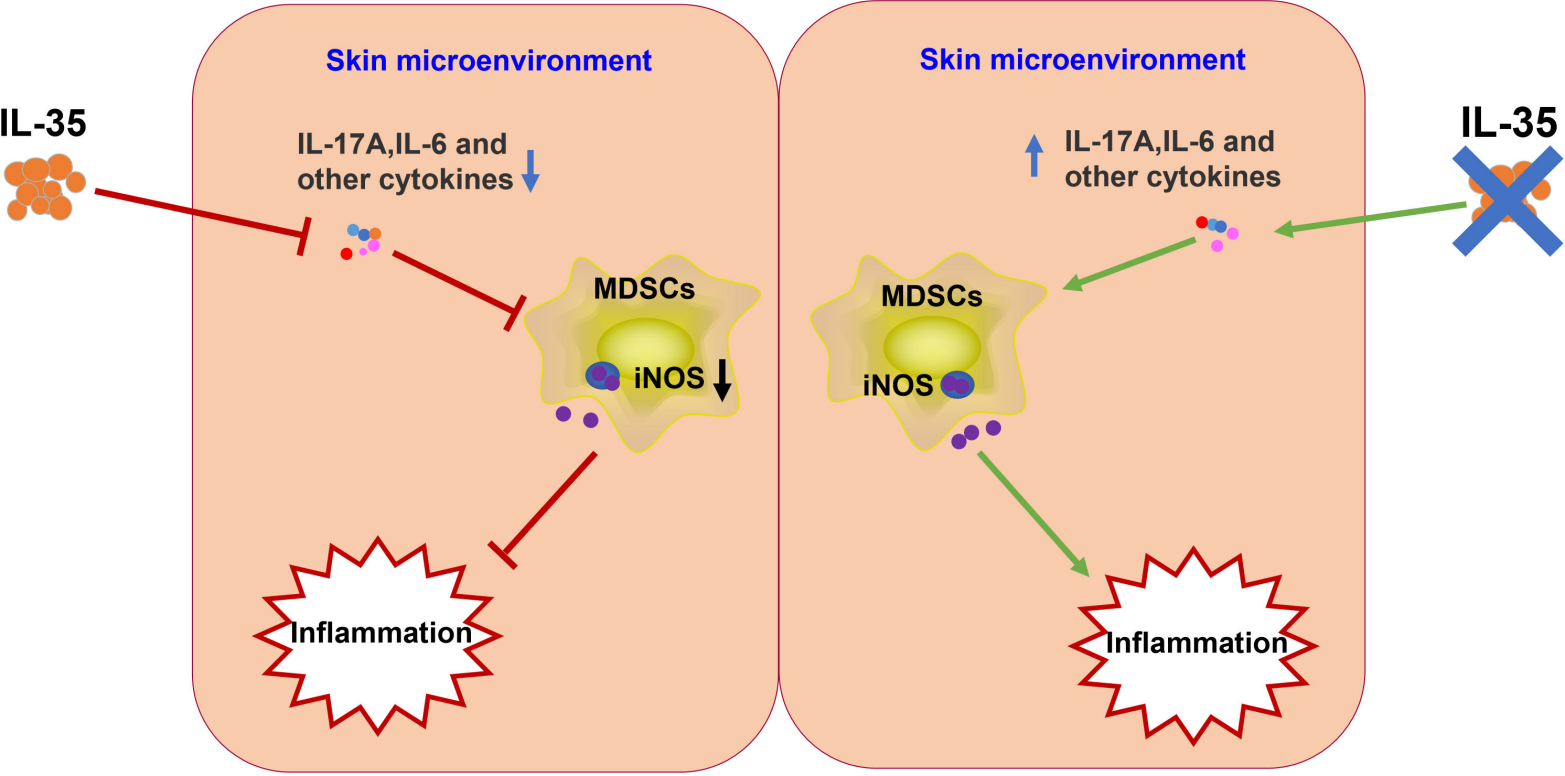
Schematic illustration of the potential mechanism underlying IL-35 amelioration of psoriasis. IL-35 inhibits proinflammatory factors in the skin microenvironment. These proinflammatory factors induce the differentiation and recruitment of MDSCs. Therefore, IL-35 may indirectly inhibit iNOS secretion from MDSCs. IL, interleukin; iNOS, inducible nitric oxide synthase; MDSC, myeloid-derived suppressor cell.

We describe the role of MDSCs in the pathogenesis of psoriasis. IL-35 attenuated psoriasis-like skin inflammation in psoriatic mice by modulating the accumulation of MDSCs. Mechanistic studies have shown that IL-35 reduces the severity of psoriasis in mice by inhibiting proinflammatory cytokines in the skin microenvironment, suppressing the recruitment of MDSCs, and reducing the population of iNOS-expressing MDSCs. We hypothesize that IL-35 may inhibit the recruitment of MDSCs and the ability of MDSCs to express iNOS, by modulating inflammatory factors in the skin microenvironment (**Figure 10**). However, our research has not deeply explored the specific signaling pathway through which IL-35 regulates the expression of iNOS in MDSCs, which will also be the focus of our later in-depth research. We conclude that IL-35 plays a critical role in the pathogenesis of psoriasis by regulating iNOS-expressing MDSCs, highlighting IL-35 as a novel therapeutic strategy for psoriasis or other cutaneous inflammatory diseases.

## Data availability statement

The raw data supporting the conclusions of this article will be made available by the authors, without undue reservation.

## Ethics statement

The studies involving human participants were reviewed and approved by Medical Ethics Committee of Jining Medical University (JNMC-2022-YX-004). The patients/participants provided their written informed consent to participate in this study. The animal study was reviewed and approved by The Institutional Animal Ethics Committee of Jining Medical University(approval number: 2018-JC-003).

## Author contributions

JZ: conceptualization, methodology, investigation, visualization, funding acquisition, and writing - original draft preparation. YZ: methodology and formal analysis. ZY: data curation. DC: formal analysis. HZ: data curation. LW: data curation. CLiu: formal analysis. FY: investigation. CL: investigation. GD: investigation. CW: software. DS: conceptualization, project administration, and funding acquisition. HX: conceptualization, project administration, and funding acquisition. All authors contributed to the article and approved the submitted version.
