# Supplementary material for: Inducible nitric oxide synthase-expressing myeloid-derived suppressor cells regulated by interleukin 35 contribute to the pathogenesis of psoriasis
Source: Front Immunol. 2023 Mar 9;14:1091541. doi: 10.3389/fimmu.2023.1091541 (PMC10034090; doi:10.3389/fimmu.2023.1091541)
Supplement: Supplementary file 1 [file DataSheet_1.docx]

Supplementary Material

# Supplementary Materials and Methods

## Construction of an in vitro psoriatic model

Human keratinocyte cell line (HaCaT) cells (provided by Professor Jiong Li from the State Key Laboratory of Biotherapy and Cancer Center, Chengdu, China) were maintained in Dulbecco’s Modified Eagle Medium (Thermo Fisher Scientific, Waltham, MA, USA) supplemented with 10% fetal bovine serum (Thermo Fisher Scientific), 100 μg/mL streptomycin (Thermo Fisher Scientific), and 100 U/mL penicillin (Thermo Fisher Scientific). Inflammation was induced in HaCaT cells following their stimulation with M5 (a mixture of five proinflammatory cytokines: tumor necrosis factor-alpha, interleukin [IL]-17A, IL-22, IL-1a, and oncostatin-M [10 ng/mL each]; ProSpec, East Brunswick, NJ, USA), thereby mimicking various characteristics of psoriasis(1) . Briefly, HaCaT cells were seeded in 6-well plates and cultured for 12 hours. After 12 hours of culture, cells were treated with different concentrations of IL-35 (0/50/100/200 ng/mL) and stimulated with M5, and the secretion of IL-6 and C-X-C motif chemokine ligand 8 (CXCL8) was determined. To determine the levels of secreted IL-6 and CXCL8, culture supernatants were collected at 24, 48, and 72 hours post-stimulation.

## Challenges with IL-35 and anti-p35 monoclonal antibody treatment in the imiquimod (IMQ)-induced psoriasis mouse model

Female C57BL/6 mice (8 weeks old) were administered a daily dose of 62.5 mg IMQ cream on the back-skin of shaved mice for 7 d (days 1–7). For the therapeutic potential of IL-35, we administered IL-35 (5 μg) one day before (day 0) the establishment of the IMQ-induced psoriasis mouse model, while three additional injections were administered every other day (days 2, 4, and 6) during the establishment of the model. To study the effect of blocking IL-35 during the process of establishing a psoriasis mouse model, rat anti-murine anti-IL-12p35 mAb (C18.2) or rat IgG 2a was administered i.p. (250 μg/mouse) on days 0 and 4 (2, 3). Animals were then euthanized on day 8, and samples of psoriatic lesion tissues were collected.

## K14-VEGF-A-Tg mouse psoriasis model and therapy

All animal experiments were performed in accordance with the National Research Council's Guide for the Care and Use of Laboratory Animals. K14-VEGF-A-Tg homozygous mice overexpressing VEGF in the epidermis were used for the experiments, and these mice spontaneously developed a chronic inflammatory skin disease with many features similar to those observed in human psoriasis (4). These mice were provided by Professor Jiong Li from the State Key Laboratory of Biotherapy and Cancer Center (Chengdu, China). K14-VEGF-A-Tg homozygous mice (10 weeks old, psoriasis-like symptoms began to appear) were randomly assigned to two groups (n = 5 per group). Each mouse was subjected to a total of 10 intravenous injections of either phosphate-buffered saline (PBS; control) or 5 μg IL-35 recombinant protein (10705-H02H, Sino Biological, Beijing, China) every other day. At 24 h after the last injection, the animals were anaesthetized using 50 μL of chloral hydrate (10%; intraperitoneal injection, Tianjin Regent Chemicals Co., LTD., Tianjin, China) for macroscopic photography. The animals were then euthanized, and psoriatic lesion tissues were collected. All procedures were performed at Jining Medical University.

# Supplementary Figures

**
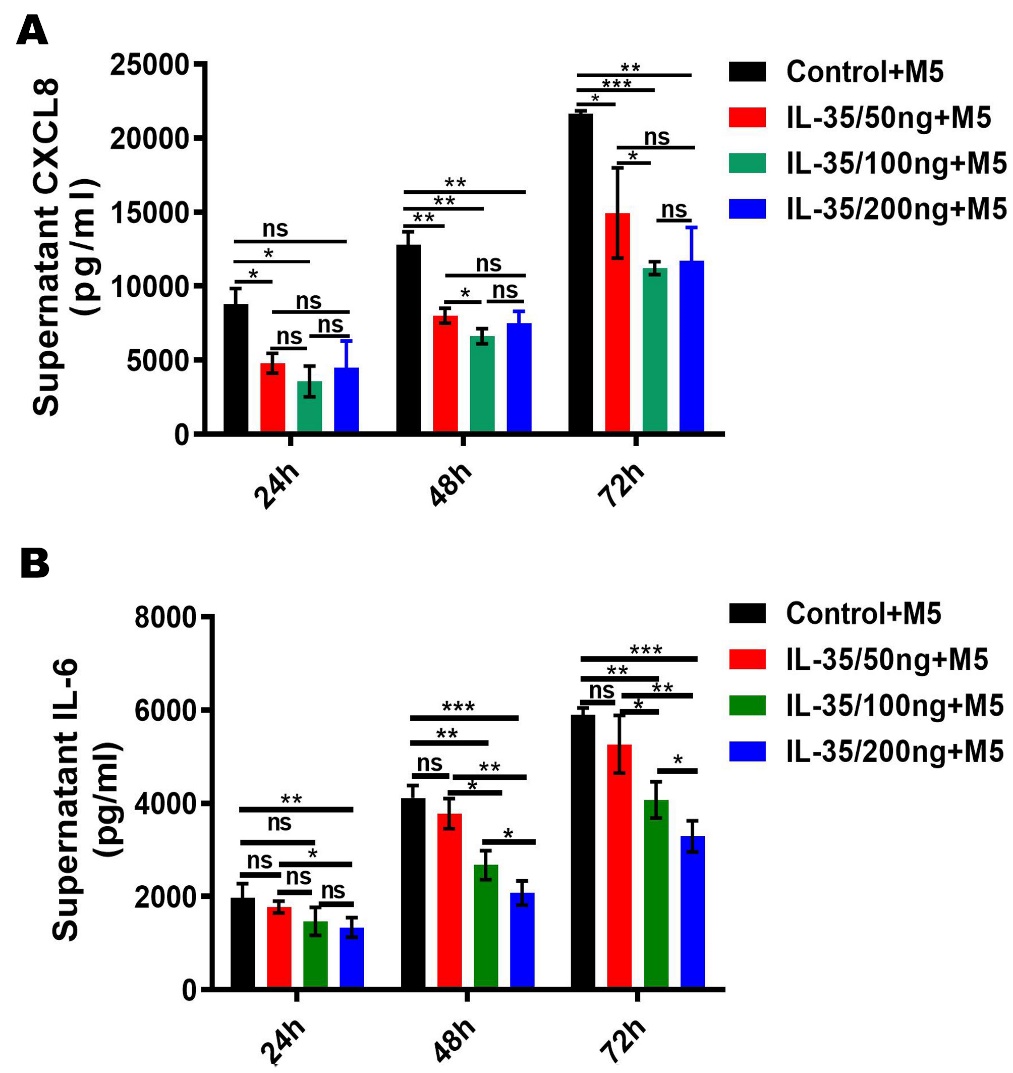
**

**Figure S1. IL-35 recombinant protein affects the production of IL-6 and CXCL8 in M5-stimulated HaCaT cells**. HaCaT cells were seeded in 6-well plates and cultured for 12 hours. After 12 hours of culture, cells were treated with different concentrations of IL-35 (0/50/100/200 ng/mL) and stimulated with M5 (10 ng/mL). The production of IL-6 and CXCL8 was determined by ELISA. Expression of **(A)** CXCL8 and **(B)** IL-6. **P* < 0.05, ***P* < 0.01, ****P* < 0.001. Data are representative of three independent experiments. CXCL8, C-X-C motif chemokine ligand 8; ELISA, enzyme-linked immunosorbent assay; HaCaT, human keratinocyte cell line; IL, interleukin.

**
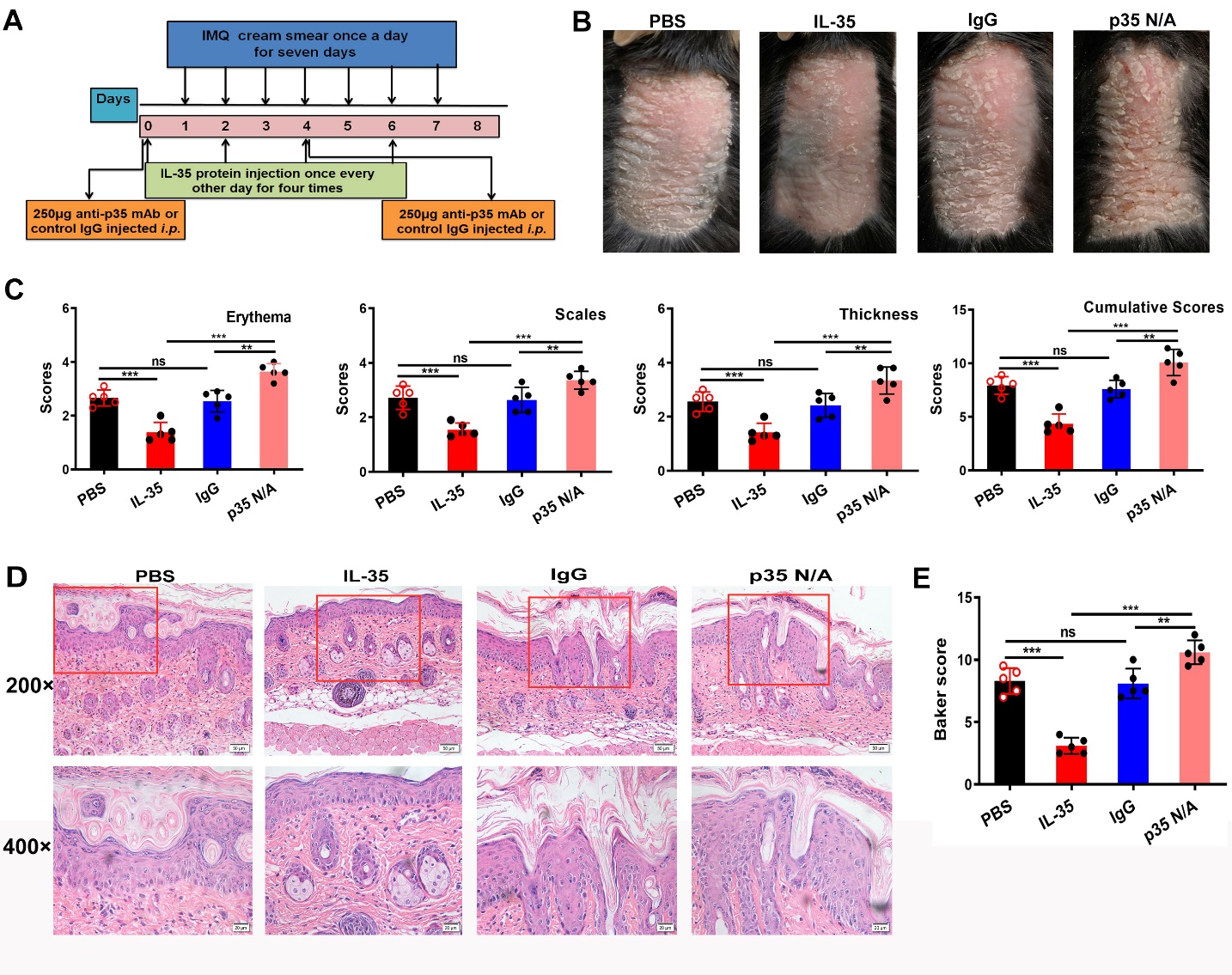
**

**Figure S2.** **Anti-mouse p35 neutralizing antibodies exacerbated symptoms in IMQ-induced psoriasis mice. (A)** Protocol for IL-35 and p35 neutralizing antibody treatment of IMQ-induced psoriasis. **(B)** Representative phenotype of murine skin after IL-35 and p35 neutralizing antibody treatment. **(C)** PASI scoring of erythema, scaling, and thickness, and the cumulative scores for different groups. **(D)** H&E-stained skin sections (200× and 400× magnification; the 400× image is the enlarged image in the box). **(E)** Pathological scores of skin sections (N = 5) using the Baker scoring system. Data are presented as means ± standard deviation. ***P* < 0.01, ****P* < 0.001. H&E, hematoxylin and eosin; IL, interleukin; IMQ, imiquimod; ns, not significant; PASI, Psoriasis Area and Severity Index.

**
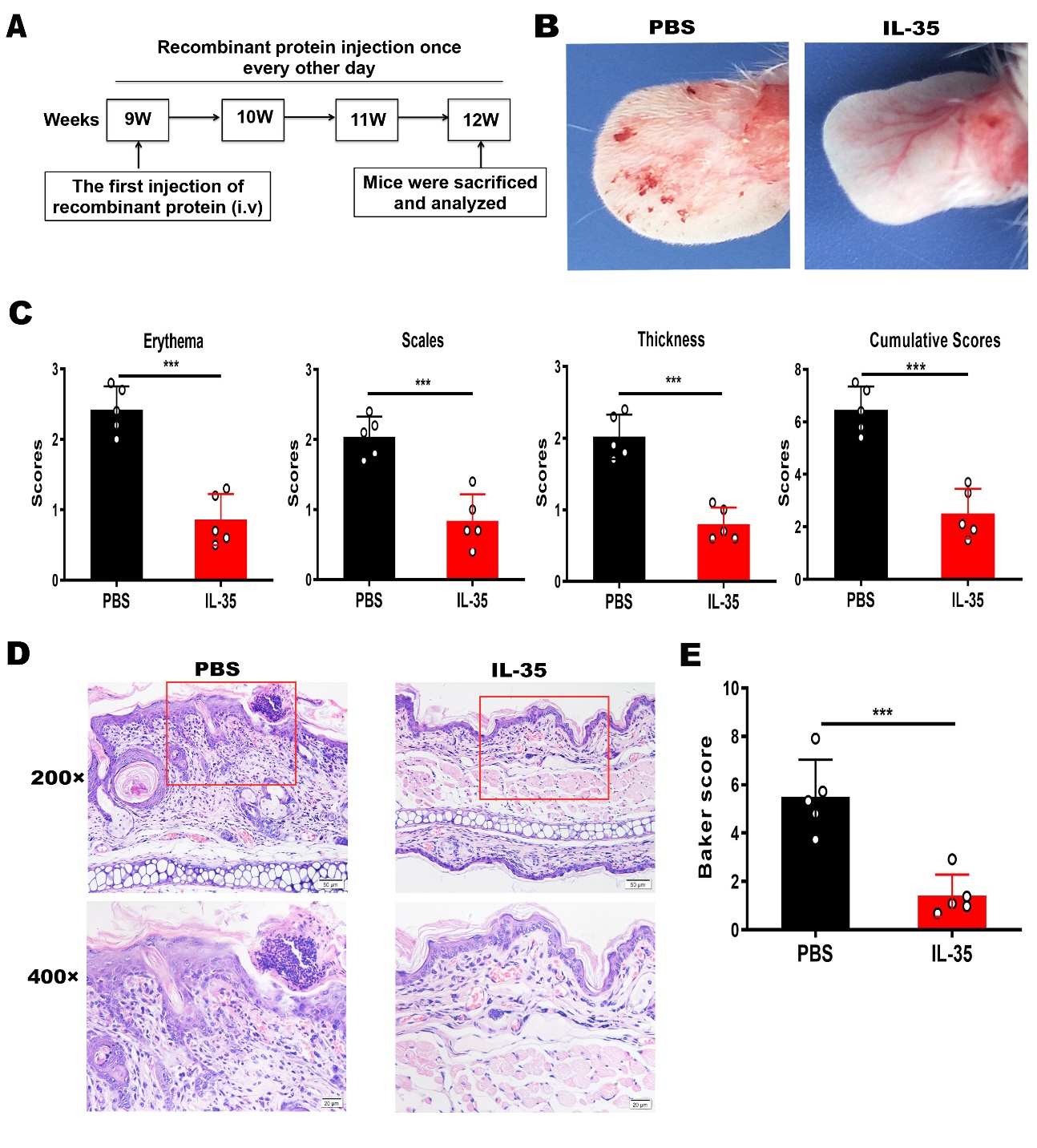
**

**Figure S3. IL-35 recombinant protein alleviated pathological characteristics of psoriatic lesions in K14-VEGF-A-Tg mice.** (a) Schedule of the therapeutic delivery of IL-35. (b) The phenotype of the mouse ear after therapy (n = 5). (c) Individual psoriasis area and severity index (PASI) scores of erythema, scaling, and thickness as well as cumulative PASI scores. (d) Hematoxylin and eosin (H&E) staining of the murine ear skin (original magnification 200× and 400×, the 400× image is the enlarged image in the box in the 200× image). (e) Pathological score of ear sections using the Baker scoring system. Columns represent the mean, while the bars represent the standard deviation (SD). ***p < 0.001. The data are representative of three independent experiments.


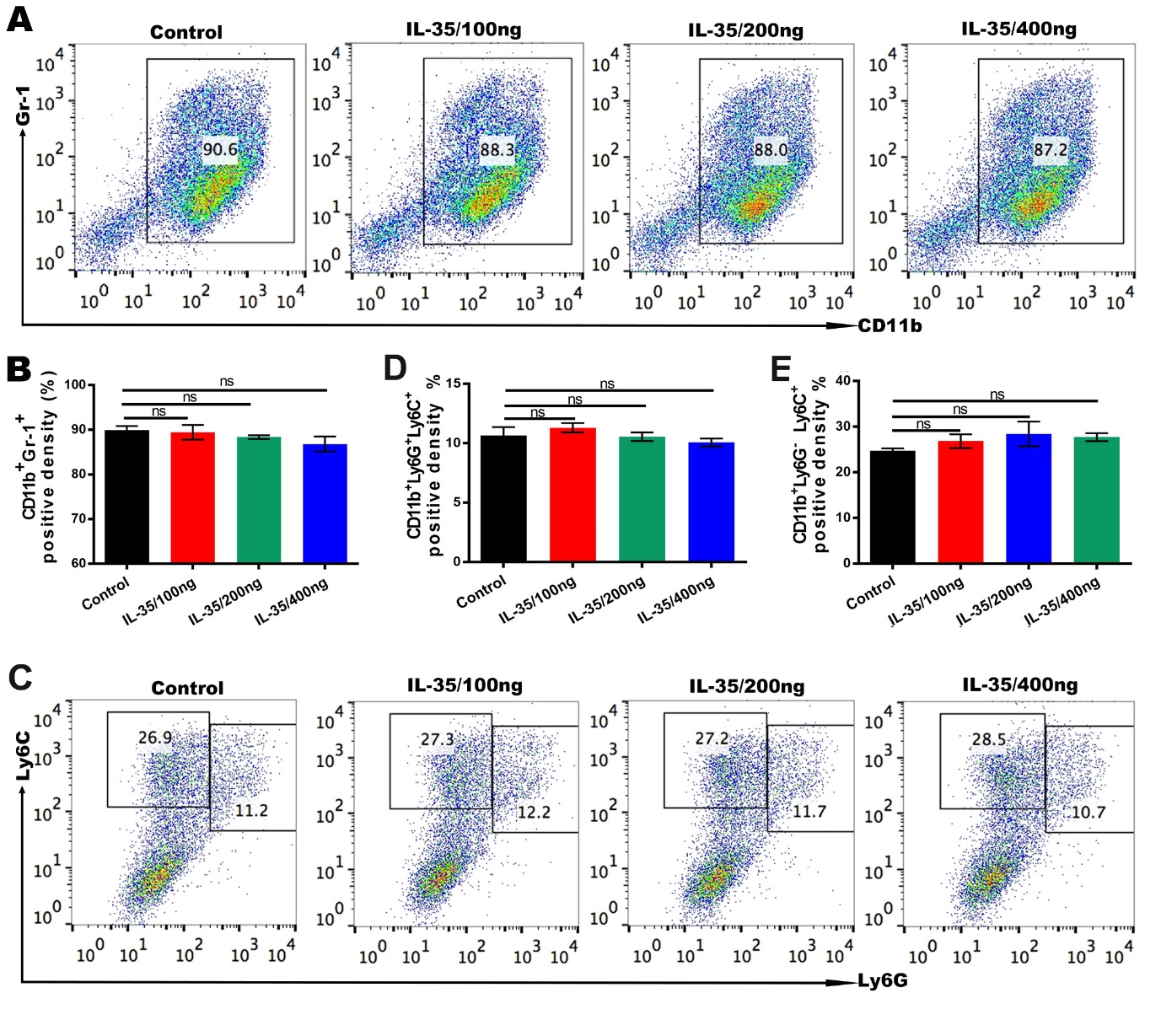


**Figure S4. Effect of IL-35 on the differentiation of myeloid-derived progenitor cells** (**MDSCs) induced by granulocyte-macrophage colony-stimulating factor (GM-CSF) and IL-6 *in vitro*.** (a–e) Bone marrow cells pre-treated with different concentrations of IL-35 (100, 200, and 400 ng/mL) or vehicle and were cultured with murine GM-CSF (40 ng/mL) and IL-6 (40 ng/mL). After four days, the numbers of CD11b^+^Gr-1^+^ MDSCs (a, b), CD11b^+^Ly6G^+^Ly6C^low^ granulocytic MDSCs (G-MDSCs) (c, d), and CD11b^+^Ly6G^-^Ly6C^high^ M-MDSCs (c, e) were analyzed using fluorescence-activated cell sorting (FACS). Data are representative of three biological replicates, each using three technical replicates. ns, no significance.

# References

1. Guilloteau K, Paris I, Pedretti N, Boniface K, Juchaux F, Huguier V, et al. Skin Inflammation Induced by the Synergistic Action of IL-17A, IL-22, Oncostatin M, IL-1{alpha}, and TNF-{alpha} Recapitulates Some Features of Psoriasis. *J Immunol* (2010) 184(9):5263-5270. doi: 10.4049/jimmunol.0902464

2. Meyts I, Hellings PW, Hens G, Vanaudenaerde BM, Verbinnen B, Heremans H, et al. IL-12 contributes to allergen-induced airway inflammation in experimental asthma. *J Immunol* (2006) 177(9):6460-70. doi: 10.4049/jimmunol.177.9.6460

3. Bitsaktsis C, Babadjanova Z, Gosselin EJ. In vivo mechanisms involved in enhanced protection utilizing an Fc receptor-targeted mucosal vaccine platform in a bacterial vaccine and challenge model. *Infect Immun* (2015) 83(1):77-89. doi: 10.1128/IAI.02289-14

4. Xia YP, Li B, Hylton D, Detmar M, Yancopoulos GD, Rudge JS. Transgenic delivery of VEGF to mouse skin leads to an inflammatory condition resembling human psoriasis. *Blood* (2003) 102(1):161-8. doi: 10.1182/blood-2002-12-3793
